# Method for Stress Assessment of Endosymbiotic Algae in *Paramecium bursaria* as a Model System for Endosymbiosis

**DOI:** 10.3390/microorganisms10061248

**Published:** 2022-06-18

**Authors:** Toshiyuki Takahashi

**Affiliations:** Department of Chemical Science and Engineering, National Institute of Technology (KOSEN), Miyakonojo College, Miyazaki 885-8567, Japan; mttaka@cc.miyakonojo-nct.ac.jp; Tel.: +81-986-47-1219

**Keywords:** microcapillary flow cytometry, fluorescence, chlorophyll, herbicide, paraquat

## Abstract

Endosymbiosis between heterotrophic host and microalga often breaks down because of environmental conditions, such as temperature change and exposure to toxic substances. By the time of the apparent breakdown of endosymbiosis, it is often too late for the endosymbiotic system to recover. In this study, I developed a technique for the stress assessment of endosymbiotic algae using *Paramecium bursaria* as an endosymbiosis model, after treatment with the herbicide paraquat, an endosymbiotic collapse inducer. Microcapillary flow cytometry was employed to evaluate a large number of cells in an approach that is more rapid than microscopy evaluation. In the assay, red fluorescence of the chlorophyll reflected the number of endosymbionts within the host cell, while yellow fluorescence fluctuated in response to the deteriorating viability of the endosymbiont under stress. Hence, the yellow/red fluorescence intensity ratio can be used as an algal stress index independent of the algal number. An optical evaluation revealed that the viability of the endosymbiotic algae within the host cell decreased after treatment with paraquat and that the remaining endosymbionts were exposed to high stress. The devised assay is a potential environmental monitoring method, applicable not only to *P. bursaria* but also to multicellular symbiotic units, such as corals.

## 1. Introduction

Protists from the genus *Paramecium* (*Alveolata*, *Ciliophora*) are used as model organisms in various research fields [1,2,3,4,5,6]. Paramecia are unicellular organisms with thousands of cilia [1,2,3,4]. 

Within the genus *Paramecium*, the green ciliate *Paramecium bursaria* has been used as a model organism for endosymbiosis that has driven the cellular evolution of eukaryotes [7,8,9,10,11,12,13,14]. Numerous endosymbiotic algae, similar to the green alga *Chlorella* (*Trebouxiophyceae*, *Chlorophyta*, and *Viridiplantae*), establish symbiosis within the paramecium host [15]. The paramecium host cell indirectly utilizes the photosynthetic products that are released by endosymbiotic algae and consequently becomes tolerant to starvation, such as the depletion of bacteria [16,17,18,19]. Therefore, the endosymbionts provide the paramecium host with additional features that support its survival. 

Endosymbiotic algae can be artificially removed from the paramecium host by using certain experimental procedures, such as treatments with herbicides, protein synthesis inhibitors or other chemicals, and continuous incubation in darkness [12,17,19,20,21,22]. However, even when the endosymbiotic algae are removed, the host cell continues to live and divides asexually, by cell division, under appropriate nutritional conditions with sufficient bacteria to feed on [17,19]. The alga-free host can also reproduce sexually by conjugation [17,19]. 

Endosymbiotic relationships between heterotrophic cells and autotrophic microalgae have been observed not only in *P. bursaria* but also in some multicellular organisms, such as *Hydra viridissima* [23,24,25], coral [23], anemone, sponge [26,27,28,29,30], and the nudibranch *Melibe engeli* [31]. The removal of endosymbiotic algae upon exposure to herbicides and certain toxic substances, and the breakdown of endosymbiosis are driven by the differential sensitivity of the host cell and the endosymbiont to these chemicals [21,32]. Therefore, the integrity of an integrative cell consisting of the host cell and the endosymbiont must be evaluated, not only from the standpoint of the cellular activity of the host cell, but also considering the integrity of the endosymbiont. However, while the morphology and movement of the host can be easily observed, the integrity of the endosymbiotic algae within the host cell is difficult to assess. This precludes timely observation of the distress of endosymbiotic algae, e.g., that which is induced by environmental pollution or environmental changes. Coral bleaching events, which lead to the death of the cnidarian host and the collapse of the reef ecosystem are an example consequence of the removal of endosymbiotic dinoflagellate alga (*Symbiodinium*) from the host cell [33,34,35,36,37,38]. Hence, the development of a technology for monitoring the endosymbiotic state as an integrated unit composed of the host cell and endosymbiont before the final and apparent breakdown of endosymbiosis is urgently needed from the environmental monitoring perspective. 

The aim of the current study was to develop a method for evaluating the viability of a symbiotic unit comprising *P. bursaria*. Although microscope-based methods are used to evaluate host morphology and movement, they cannot be used to quantitatively assess the state of endosymbiotic algae in the host cell. Considering differences in the sensitivity of the endosymbiont and the host to toxic compounds, a method is needed for a rapid and quantitative evaluation of the status of many cells. Accordingly, the method that is developed herein employs microcapillary flow cytometry (FCM), which can be used to analyze relatively large cells, such as *P. bursaria* [19,39,40]. In addition to the evaluation of the endosymbiotic status of *P. bursaria* as an integrative cell, i.e., whether the *P. bursaria* host harbors a sufficient number of endosymbiotic algae to maintain the endosymbiosis, the devised method can be used to quantify the stress level of endosymbiotic algae in the host cells following treatment with a herbicide as an endosymbiotic-collapse inducer. The method could be adapted for environmental monitoring applications to evaluate the endosymbiotic status of different endosymbiotic organisms and to detect the crisis of endosymbiosis. 

## 2. Materials and Methods

### 2.1. P. bursaria Strain and Culture Conditions

*P. bursaria* syngen I (AS-2, mating type IV) collected from the Ashida Kawa River (Hiroshima prefecture, Japan) was used. The paramecia were cultured in lettuce infusion [20] containing *Klebsiella pneumoniae* as food under an LD cycle (12 h light/12 h dark) with ca. 1100 lux (ca. 17 μmol·m^−2^·s^−1^ as photosynthetic photon flux density [PPFD]) natural white fluorescent light and 23 ± 2 °C. *P. bursaria* in logarithmic to the stationary phase of growth. The paramecia were then collected and used for subsequent experiments.

### 2.2. Specimen Preparation and Light Microscopy

The paramecia were harvested and fixed in 5% (*v*/*v*) formalin. A bright field image of the prepared specimens was analyzed using a light microscope (Labolux Jr. Series KN-50TC, Kyowa Optical Co. Ltd., Sagamihara, Kanagawa, Japan) that was equipped with a digital camera (AR-D300C, Arms System Co. Ltd., Tokyo, Japan). 

### 2.3. Detection of Intact P. bursaria Using Microcapillary FCM

**Figure 1:**
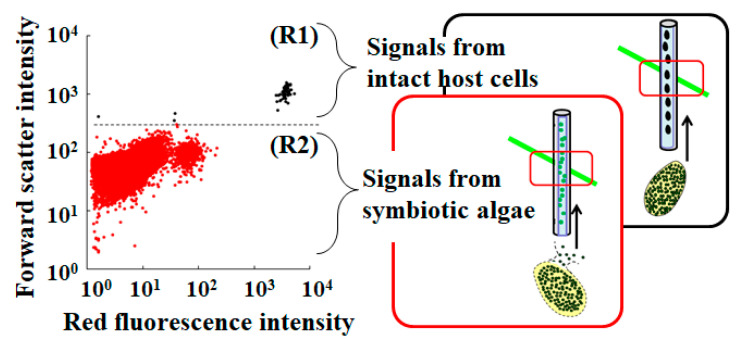
An example scatter plot of *P. bursaria* analyzed using microcapillary FCM. Signals in the R1 region with high forward scatter intensity and high red fluorescence intensity correspond to intact *P. bursaria* cells, while signals in the R2 region with low forward scatter intensity and low red fluorescence intensity correspond to endosymbionts released from broken *P. bursaria* cells.

To detect and evaluate the intact *P. bursaria* host cells in a test sample, a microcapillary flow cytometer that was equipped with a green laser operating at 532 nm (Muse^TM^ Cell Analyzer, Merck Millipore Corp., Burlington, MA, USA) was used, as described earlier [19,39]. *P. bursaria* fixed in 5% (*v*/*v*) formalin was analyzed at flow rates of 0.59 µL/s. Several optical properties of *P. bursaria* cells that passed through a rectangular capillary with a 100-µm round bore were analyzed. Forward scatter signals (forward scatter-height intensity) were collected to confirm the cell size. Simultaneously, chlorophyll fluorescence was detected in the red fluorescence channel using a 680/30 nm band-pass filter. In addition to the red fluorescence channel (red fluorescence intensity), a yellow fluorescence channel (576/28 nm band-pass filter) was simultaneously used. To obtain sufficient data, FCM measurements were repeated 3–20 times. Individual signals were extracted from the FCM data for each generated FCS3.0 file using FCSExtract utility ver. 1.02 (Stowers Institute for Medical Research, Kansas City, MO, USA). The data were re-analyzed using standard spreadsheet software (Excel, Microsoft Corp., Redmond, WA, USA). In microcapillary FCM, the intact *P. bursaria* host cells as target cells pass through a 100-μm-wide capillary for analysis, which allows passage of their paramecia [19,39]. If the *P. bursaria* host cells are not disrupted during the measurement, information about them is obtained [39]. Conversely, disruption of the host cell prior to analysis allows collection of information on endosymbiotic algae that are released from the crushed host cells [40]. If the FCM data of *P. bursaria* are expressed in a scatter plot of forward scatter intensity vs. red fluorescence intensity, two distinct populations are detected (R1, representing the intact *P. bursaria* cells, and R2, representing the endosymbionts that are released from the broken *P. bursaria* cells) (Figure 1), as described earlier [39]. 

### 2.4. Treatment of P. bursaria with Paraquat (PQ) Herbicide

PQ (methyl viologen, C_12_H_14_C_l2_N_2_) was used to destabilize the microalgal endosymbiosis with *P. bursaria* [17,20,32,39,41,42,43]. Prior to the experiment, *P. bursaria* was cultured in fresh lettuce medium containing *K. pneumoniae* that was supplemented with 0.1 μg/mL of PQ for 1–7 d under an LD cycle at ca. 1100 lux (ca. 17 μmol·m^−2^·s^−1^ as PPFD) natural white fluorescence light and 23 ± 2 °C (initial density: 20 paramecia/mL) [39]. After PQ treatment, the paramecia were collected and fixed in 5% (*v*/*v*) formalin. The fixed paramecia were used for microscopic observations and flow cytometric analyses. For FCM analysis, signals in the R 1 region that were derived from the intact host cells were acquired and analyzed as described in Section 2.3. Results from duplicate and triplicate experiments with PQ were used for the FCM analysis. 

### 2.5. FCM Evaluation of the Stress Status of Endosymbiotic Algae in P. bursaria

To evaluate the cellular status of endosymbiotic algae in the *P. bursaria* host cell, the red and yellow fluorescence intensities were evaluated using signals that were derived from the intact *P. bursaria* host cells (Figure 1). The stress of endosymbiotic algae was defined as the variation in parameters related to chlorophyll that was detected using FCM when compared to the control condition without herbicide. The fluorescence intensities and scatter plot distribution patterns of PQ-treated and untreated *P. bursaria* were compared. 

To assess the stress status of endosymbiotic algae in host cells in the untreated control and in PQ-treated samples, the yellow/red fluorescence intensity ratios of individual intact *P. bursaria* host cells were determined. After a statistical analysis of quantiles, such as a sample minimum, lower quartile, median, upper quartile and maximum using Microsoft Excel, a box-and-whisker plot analysis was performed using Microsoft Excel functions.

## 3. Results

### 3.1. Effects of the Herbicide PQ on Microalga Endosymbiosis with P. bursaria and Endosymbiosis Characterization Using FCM

#### 3.1.1. Detection of Red Fluorescence Signals from *P. bursaria* Cells Using FCM

**Figure 2:**
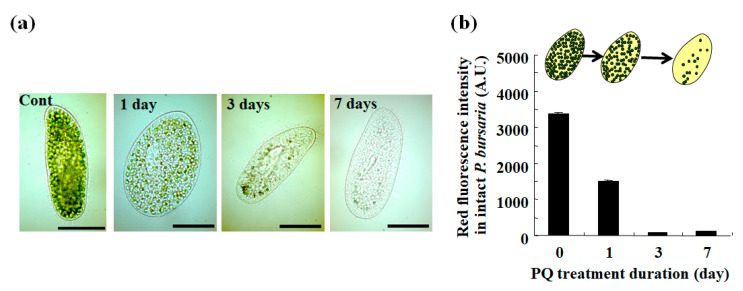
*P. bursaria* treated with the herbicide paraquat (PQ). (**a**) Photographs of representative *P. bursaria* cells treated or not (Cont) with 0.1 μg/mL of PQ for up to 7 d. Scale bar, 50 μm. (**b**) *P. bursaria* treated or not with PQ were analyzed using microcapillary FCM. The results are expressed as mean ± standard deviation (*n* > 320 signals [320 cells of intact *P. bursaria*]) in a bar graph of red fluorescence intensity.

As previously reported [17,20,32,39,40,41,42,43], the PQ exposure of *P. bursaria* markedly reduced the number of endosymbiotic algae in the host cell within a few days of treatment (Figure 2a). The *P. bursaria* host cells, treated or not with PQ, were collected and analyzed by microcapillary FMC. Chlorophyll, when excited by an appropriate excitation light, emits red fluorescence. Then, the FCM signals that are derived from the host cells were acquired (Figure 1), based on the red fluorescence intensity of the chlorophyll of the endosymbiotic algae in the host cell. The signal detection rate in the R1 region, representing signals from the intact *P. bursaria* cells was low in the FCM measurements using unfixed *P. bursaria*, while the signal detection in the R1 region increased in fixed cells (data not shown). Therefore, the subsequent FCM measurements were performed using fixed *P. bursaria* cells. As a result of the FCM measurements, the red fluorescence intensity of the *P. bursaria* host cells decreased depending on the duration of the PQ treatment (Figure 2b). As shown in the microscopic images in Figure 2a, the decrease in red fluorescence intensity was related to a notable reduction in the number of endosymbiotic algae in the individual host cells. However, the FCM red fluorescence intensity information was difficult to interpret beyond the cell appearance in microscopic images. 

#### 3.1.2. Detection of Yellow Fluorescence Signals from *P*. *bursaria* Cells Using FCM

The number of endosymbiotic algae in each host cell fluctuates not only as a result of cytotoxicity upon certain chemical exposure but also throughout the *P. bursaria* cell cycle [19,44], and depending on nutrient availability and other factors, such as culture conditions. Hence, the information that is gleaned from changes in the red fluorescence intensity cannot serve as an assessment of the vitality of endosymbiotic algae because it is directly dependent on the number of endosymbiotic algae in the *P. bursaria* cell (Figure 2). Therefore, in addition to the red fluorescence intensity data, the corresponding yellow fluorescence intensity, reflecting the physiological activity of the endosymbiont, was evaluated in the current study. 

**Figure 3:**
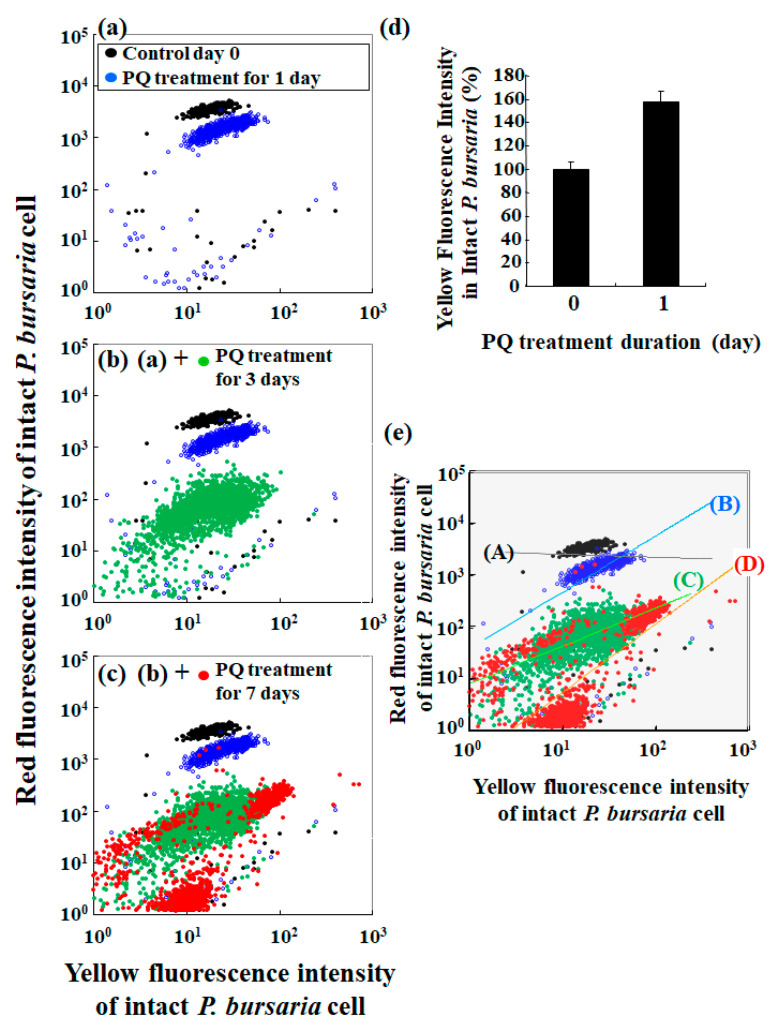
Characteristics of red and yellow fluorescence intensities of *P. bursaria* cells analyzed using FCM. (**a**–**c**) Scatter plots of yellow fluorescence intensity vs. red fluorescence intensity of *P. bursaria* cells treated or not (control, day 0) with PQ (0.1 μg/mL). (**d**) Difference in yellow fluorescence intensity of untreated and PQ-treated (1 d) *P. bursaria* cells. Data were expressed as mean ± standard error (*n* > 320 signals [320 cells of intact *P. bursaria*]). (**e**) Slop analysis of scatter plots for *P. bursaria* treated or not with PQ.

Figure 3 shows scatter plots of the red fluorescence intensity and the corresponding yellow fluorescence intensity. The yellow fluorescence of algae is related to chlorophyll integrity [45,46,47,48], while the red fluorescence directly reflects the number of algae and an algal population size [14,49,50,51]. Comparison of the scatter plot pattern of untreated controls and those of cells that were treated with PQ revealed changes after 1 d of PQ treatment (Figure 3a). Furthermore, the plot pattern was clearly different from that for the control condition, depending on the duration of the PQ treatment (Figure 3a–c). Specifically, the yellow fluorescence intensity of the cells that were treated with PQ for 1 d was notably greater than that of the control cells (Figure 3d). However, the increase in yellow fluorescence intensity was not pronounced after 3–7 d of PQ treatment when compared to the yellow fluorescence intensity in cells after 1 d of PQ treatment (data not shown).

A detailed analysis of the scatter plots of red and the corresponding yellow fluorescence intensities revealed different tendencies of plot distribution patterns for the control and PQ-treated samples. Accordingly, the correlation between the red and yellow fluorescence intensities in the control samples was negligible (slop (A) in Figure 3e), while there was a clear correlation between the red and yellow fluorescence intensities in the PQ-treated samples, with the slops determined by the least-squares method (slops (B) to (D) in Figure 3e).

### 3.2. The Ratio of Yellow Fluorescence Intensity to the Corresponding Red Fluorescence Intensity of P. bursaria as a Stress Index

#### 3.2.1. Fluctuation of the Stress Index throughout the Cell Cycle of *P*. *bursaria* Host

**Figure 4:**
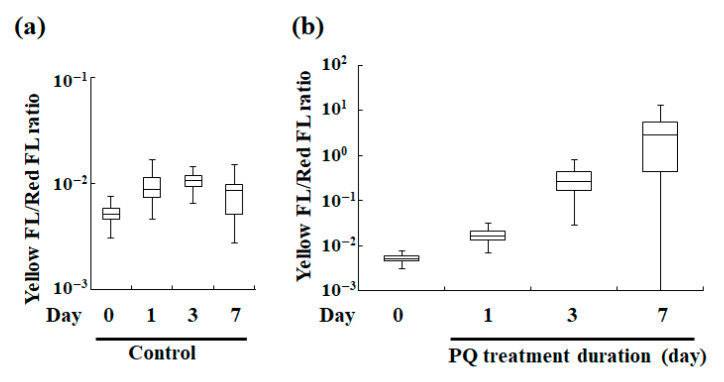
Box-and-whisker diagram analysis of ratios of the yellow fluorescence intensity (Yellow FL) to the corresponding red fluorescence intensity (Red FL) of *P. bursaria*. (**a**) Changes in the Yellow FL/Red FL ratio throughout the cell cycle of control *P. bursaria* host cell. (**b**) Changes in the Yellow FL/Red FL ratio in cells treated with PQ (0.1 μg/mL). The upper and lower error bars indicate the maximum and minimum value, respectively. A bar in each box represents the corresponding median value (*n* > 320 signals [320 intact *P. bursaria* cells]).

Since the correlation between the red and yellow fluorescence intensities depended on the degree of stress that was induced by PQ (Figure 3), the cellular stress status of the endosymbiotic algae in the host cell was next evaluated. Specifically, the ratio of yellow fluorescence intensity to the corresponding red fluorescence intensity was used as a stress index for the endosymbiotic algae in the *P. bursaria* host. Based on microphotographs (Figure 2a) and the red fluorescence intensity of *P. bursaria* that was treated with PQ (Figure 2b), the number of endosymbiotic algae in *P. bursaria* cells was directly correlated with the corresponding red fluorescence intensity of the intact *P. bursaria* host cell. To compare the cellular stress status of the endosymbiotic algae in *P. bursaria* under different experimental conditions, regardless of the difference in the number of endosymbiotic algae per host cell, the ratios of yellow fluorescence intensity to the corresponding red intensity that were derived from the individual intact *P. bursaria* host cells were calculated. To minimize the differences in the number of endosymbiotic algae under the different experimental conditions, the red fluorescence intensity corresponding to the number of endosymbiotic algae was the denominator, and the yellow fluorescence intensity was the numerator. Furthermore, changes in the ratio of yellow fluorescence intensity to the red fluorescence intensity were also examined throughout the cell cycle of *P. bursaria* host cells in untreated *P. bursaria* host cells (Figure 4a). 

Figure 4 shows the box-and-whisker diagram analysis of the ratios. Figure 4a shows the ratio of yellow fluorescence intensity to red fluorescence intensity of the endosymbiotic algae in *P. bursaria* without PQ treatment, but in different cell cycle phases of the host cell, thus reflecting changes in cell stress status during the cell cycle of the host cell. Although some variation in the yellow/red fluorescence intensity ratio (a stress indicator of endosymbiotic algae in *P. bursaria*) during the cell cycle transition of the host cell was apparent, the variation was nearly constant within the error range (Figure 4a).

#### 3.2.2. Evaluation of Stress Index of Endosymbionts in *P*. *bursaria* Treated with PQ

The yellow/red fluorescence intensity ratios of the PQ-treated and untreated (control) cells were compared (Figure 4b). The ratio increased even after 1 d of PQ treatment compared with that in the control. Further, the ratio value notably increased with the lengthening of the PQ treatment. As noted in Section 3.2.1, the vertical axis representing the yellow/red fluorescence intensity ratio in the box-and-whisker diagram, but not the yellow or red fluorescence, is independent of the number of endosymbiotic algae in a *P. bursaria* host cell. Therefore, the yellow/red fluorescence intensity ratio represents the intensity of cellular stress that is experienced by an individual endosymbiont cell. The data indicated that the viability of the endosymbiotic algae deteriorated depending on the duration of the PQ treatment.

**Figure 5:**
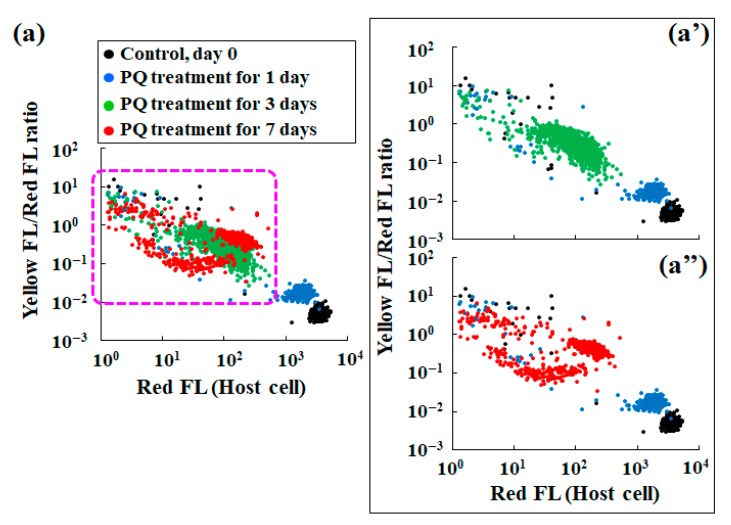
Scatter plots of the red fluorescence intensity (Red FL) vs. the ratio of yellow fluorescence intensity (Yellow FL) to the corresponding Red FL of *P. bursaria* treated or untreated (control, day 0) with PQ (0.1 μg/mL). (**a**) Scatter plots for the control and PQ-treated (1–7 d) cells. Panel (**a’**) shows all scatter plots except for the data for the 7 d PQ treatment, and panel (**a’’**) shows all scatter plots except for the data for the 3 d PQ treatment.

Figure 5 presents scatter plots that summarize the yellow/red fluorescence ratio, i.e., the average stress index of an individual endosymbiotic alga (Figure 4b), and the red fluorescence intensity reflecting the number of endosymbionts in each host cell (Figure 2b). While the distribution of the data points for the control conditions is very narrow and constant, the distribution of data points for the PQ treatment conditions differs depending on the duration of the PQ treatment. For example, the data point distribution for 1 d of PQ exposure simultaneously indicates an increase in stress and a slight decrease in the number of endosymbiotic algae, compared to those in the control. Similar to the distribution pattern for the control, the distribution pattern for 1 d of PQ treatment was constant (Figure 5a). However, the plot distribution patterns for the other test conditions (≥3 d of PQ treatment) varied considerably for individual *P. bursaria* host cells. Although there was some variation in the scatter plots (Figure 5a–a”), the longer the duration of PQ treatment, the lower the number of endosymbiotic algae in each host cell and the greater the stress index of the endosymbiotic algae that remained in each host cell.

## 4. Discussion

### 4.1. Characteristics of the Red and Yellow Fluorescence Intensity Signals from P. bursaria Obtained Using FCM

This study focused on developing a method for evaluating the cellular health integrity of *P. bursaria* that would be superior to the visual inspection method. The new method is based on the evaluation of endosymbiosis using microcapillary FCM. The herbicide PQ was used to destabilize the endosymbiotic relationship between the *P. bursaria* host and the endosymbiotic algae. The PQ treatment of *P. bursaria* induced the readily observable destabilization and breakdown of the microalga endosymbiosis with *P. bursaria*, resulting in a reduction in the number of endosymbionts in the host cell (Figure 2a). 

**Figure 6:**
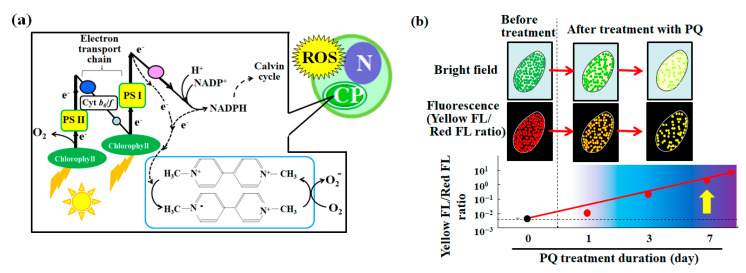
The effect of the herbicide PQ on photosynthesis and the evaluation of endosymbiotic algal vitality in *P. bursaria* using the yellow/red fluorescence intensity ratio. (**a**) Mechanism of photosynthesis-related PQ toxicity and the associated reactive oxygen species (ROS) production [52]. The acronyms PS, Cyt, N, and CP, respectively, denote photosystem, cytochrome, algal nucleus, and chloroplast. (**b**) Conceptual diagram of the devised method for the evaluation of the endosymbiotic algal vitality in *P. bursaria* cells using the yellow/red fluorescence intensity ratio as a stress index.

The mechanism of PQ toxicity in relation to photosynthesis is described in Figure 6a. Chlorophyll absorbs light energy in the light reaction of photosynthesis. After excitation, the electron transfer chain is involved in a series of redox reactions. Using electrons that are generated by this electron transfer chain, PQ reduces oxygen molecules, which leads to the production of superoxide as one of the reactive oxygen species [52] (Figure 6a). This induces oxidative stress and cytotoxicity, such as the decrease in the number of endosymbionts in the *P. bursaria* cell (Figure 2a). The flow cytometer that was used in the current study detected signals that were derived from the endosymbiotic algae in the *P. bursaria* host cell by focusing on the red fluorescence (Figure 2b) [19,39,40]. The intensity of red fluorescence (Figure 2b) was largely consistent with the changes in the number of endosymbiotic algae that were observed using microscopy (Figure 2a). 

As shown in the microscopic images of *P. bursaria* that was treated with PQ (Figure 2a), the red fluorescence intensity of *P. bursaria* (Figure 2b) notably decreased depending on the PQ treatment period because of the decrease in the number of endosymbiotic algae in *P. bursaria*. In addition to the red fluorescence intensity, the flow cytometer that was used in the current study also detected yellow fluorescence signals. The yellow fluorescence intensity is associated with chlorophyll denaturation and other factors [45,46,47,48]. Therefore, the maximum and potential yellow fluorescence intensity is related to the red fluorescence intensity that is emitted by chlorophyll [39,47]. In other words, the decrease in the number of endosymbionts in *P. bursaria* by the PQ treatment led to a decrease in the intensity of the red fluorescence as the potential source of the yellow fluorescence, leading to the ensuing reduction in the intensity of the yellow fluorescence. However, when the number of endosymbiotic algae in the host cell significantly differed in the reference (control) and the test condition, it was difficult to evaluate the microalgal stress solely on the basis of the yellow fluorescence intensity (Figure 3). This led me to consider the yellow/red fluorescence intensity ratio as a stress index for endosymbionts in *P. bursaria* host cells.

### 4.2. Using the Yellow/Red Fluorescence Intensity Ratio as the Stress Index for Endosymbionts in the P. bursaria Host Cell

The yellow/red fluorescence intensity ratio was characterized herein as the stress index of the endosymbiotic algae in the host cell (Figure 4) because of the observed correlation between the red and yellow fluorescence intensities depending on the PQ treatment duration (Figure 3e). To investigate the properties of the yellow/red fluorescence intensity ratio as a stress indicator and to check its validity, changes in the ratio values throughout the cell cycle of *P. bursaria* host cells without PQ treatment were first evaluated. The yellow/red fluorescence intensity ratio was nearly constant within the error range during the cell cycle transition of the host cells (Figure 4a). In contrast, the yellow/red fluorescence intensity ratio of the *P. bursaria* host cells that were treated with PQ was greater than that of the control cells (Figure 4b). Further, the ratio notably increased with the increasing duration of PQ treatment (Figure 4b). The ratio represents the degree of average stress per individual endosymbiont because the yellow/red fluorescence intensity ratio is independent of the number of endosymbiotic algae in a *P. bursaria* cell. The analysis clearly indicated that the PQ treatment of *P. bursaria* disrupted the endosymbiotic algae, so that the longer the treatment time, the more severe the effect. Hence, the yellow/red fluorescence intensity ratio can be used as an indicator of the vitality of endosymbiotic algae in the *P. bursaria* cell. 

The scatter plots of the yellow/red fluorescence intensity ratio (endosymbiont stress index) vs. the red fluorescence intensity of the *P. bursaria* cell (the number of endosymbionts in the host cell) provided additional information (Figure 5). The broad plot distribution of the data for the PQ-treated samples suggested that the host cell eliminates endosymbionts upon PQ treatment. In addition, it also shows the existence of a considerable individual variation among the *P. bursaria* cells in the removal of endosymbionts (Figure 5a’,a”). Regardless of the interpretation, it is clear that the number of endosymbiotic algae in each host cell decreased with the PQ treatment time, and that the endosymbiotic algae that were exposed to digestion in the host cell experienced a high level of stress (Figure 5). 

### 4.3. Utility of the Devised Method for the Visualization of Cellular Stress of Endosymbionts in the P. bursaria Host Cell

*P. bursaria* is a model organism for endosymbiosis, and the mechanisms for an initial establishment of microalga endosymbiosis with *P. bursaria* [7,11,13,17,20] and its maintenance are extensively researched [10,19,20,22,39,40,44,53,54,55]. Not all exosymbiotic microalgae, which are the symbiotic algae that are isolated from the host cell and cultured in an artificial culture medium, are able to reestablish endosymbiosis within the host cell. In addition to this, many microalgae that are taken up are simply digested in the host lysosomes [53]. Consequently, a limited number of algal cells succeed in establishing a stable endosymbiosis. This might be related to several factors, such as the cellular status of the microalgae, e.g., the appropriateness of the candidate microalga for endosymbiosis. These symbiosis-related factors are not fully understood. To date, studies of endosymbiosis in *P. bursaria* remain a simple microscopic confirmation of the establishment or failure of experimental re-symbiosis under a variety of experimental conditions.

Considering the above, it is important to determine whether the status of microalgae establishing a successful re-symbiosis is different from that of algae failing to establish re-symbiosis. The method that is presented in the current study could be used to evaluate the cellular stress status of endosymbiotic algae even when the number of endosymbionts within the host cell is low. Therefore, the method could be used to examine the minute changes in cellular stress status of the endosymbiont during the progression of a complex biochemical process, such as the establishment of endosymbiosis.

Photosynthesis is the most essential process to be carried out by plant and algal cells, and the method that is presented in the current study analyzes chlorophyll-dependent changes in fluorescence that are directly related to photosynthesis. Using chlorophyll fluorescence, measurements of photosynthetic efficiency, such as maximum quantum yield of photosystem II (F_v_/F_m_) have been attempted in experiments with *P. bursaria* [56]. The study revealed differences in photosynthetic efficiency between endosymbiotic algae in the host cell and exosymbiotic algae that were grown outside the host cell [56]. However, the measurement of photosynthetic efficiency was the result of the average of the test sample containing several *P. bursaria* host cells because of the difficulty of single cell measurement due to the cells’ measurement sensitivity. In contrast, the FCM measurements that are used in the current study can access information on a single cell of a *P. bursaria* host because one signal is derived from one cell in an FCM measurement (Figure 1). This is an advantage of using the FCM method over other fluorescence measurements.

When combined with various fluorescently labeled biochemical probes that are indicative of processes other than photosynthesis, the devised method could be used to precisely track fluctuations in endosymbiosis under different experimental conditions, such as the establishment of endosymbiosis, maintenance of endosymbiosis throughout the cell cycle of the host cell, and its breakdown in response to various environmental stresses.

### 4.4. Importance of Evaluating a Stress Status of Endosymbionts as an Environmental Monitoring and Chemical Safety Approach, and Its Potential for Future Applications

In addition to its use as a model organism for endosymbiosis, *P. bursaria* is also used in bioassays because of its high or singular sensitivity to several chemicals [32,57,58,59,60]. Some chemicals, e.g., herbicides, such as PQ, affect endosymbiotic algae as well as the *P. bursaria* host cell. Changes in the number of endosymbiotic algae in the host cell can be easily assessed visually as the most noticeable change. However, by the time that the number of endosymbiotic algae is reduced, an irreversible breakdown of the endosymbiotic relationship between the host cell and the endosymbiont has already occurred [33,34,35,36,37,38]. Therefore, techniques that can be used to assess the stress status of the endosymbiont within the host cell before the complete breakdown of the endosymbiotic relationship are crucial to environmental monitoring. The method for endosymbiont stress assessment that is presented in the current study could be used to evaluate not only unicellular host organism, such as *P. bursaria*, but also symbiotic and kleptoplastic units of multicellular organisms, such as corals and sea slugs when applied to tissue fragments or other cell samples. Hence, the devised method could be used as a potential and effective approach for environmental monitoring.
